# The Medical Congress of 1887

**Published:** 1885-08

**Authors:** 


### Editorial.

### THE MEDICAL CONGRESS OF 1887.

In the last number of this journal it was announced that the reorganized committee of the American Medical Association had dropped the section of oral and dental surgery from the list of sections established by the original committee. This action demands some notice on the part of dental journals. At the London meeting of 1881, our section played such an important part, it worked in such harmony with the other sections and contributed so largely to the distinguished success of that Congress, that we had no reason to doubt that, here in America, where dentistry has made such peculiarly rapid strides, where as a branch of medical science it was first organized, where the modern teaching of dentists as scientific men originated, here, among a people who claimed pre-eminence in practical advancement, our specialty would surely be given every opportunity to benefit by this convocation of all that was great in medicine. In London there was no distinction between the various sections. In free democratic America, it was anticipated that a section that would probably number more delegates than any other would be given peculiar facilities. We are happy to say that the Congress, as first organized by representative medical men, had fulfilled all expectations. It remained for a rump association to outrage respectable medicine by overturning that which had been established, and to attempt to found a World's Congress upon sectional issues. It shows a woful misconception of the character and purposes of the Congress when local quarrels and questions of personal bias are injected into the discussion thus early. They must indeed be men of narrow views who would force a world's professional assembly to take sides on a division of opinion among the physicians of the State of New York. Will Medicine as a whole never get above these petty squabbles? Will it never make good its boast of being a learned profession, and rise superior to the pot-house wrangles which engage only minds of small calibre ?

That dentists may understand the present status, it is necessary that we explain some things that may not be known to all of them.

The International Medical Congress is what its name indicates—a council of the representative medical men of the world, which meets every third year. It knows nothing of medical societies or medical politics, but at each meeting accepts the invitation of the medical profession of some country to hold its next session with them. It is never the guest of any medical society, but it expects that the whole profession of the country in which it meets will join in advancing its interests. It is not intended to be a convocation of small men, nor is it anticipated that it shall be so used as to advance individual interests. Especially does it discourage all professional demagogism. In this country the American Medical Association, as the nearest approach to a national organization that we have, assumed to issue the invitations through a committee of representative men, who, in the event of its acceptance, were directed to complete an organization. This committee visited Copenhagen, where the Congress of 1884 was held, extended the invitation, which was accepted, and the committee thereby became an organizing committee of the Congress.

They returned home and proceeded to complete their work by adopting rules, establishing sections and appointing their officers. That this work was well done there can be no question. But upon the reassembling of the American Medical Association in New Orleans, in March last, in a place remote from the great medical centres, and where the membership for the year would necessarily be made up largely of those who were not conversant with the needs and feeling of the profession, it was found that a few determined demagogues, oily of tongue and reckless of the best interests of the profession, had secured control of the Association. They did not belong to the class of men who have made medicine what it is. They were in no sense representatives of the better part of medical science, and so they had not been appointed to commanding positions. But they were determined in some way to make the Congress personally profitable to themselves, and they accordingly headed a movement which resulted in the undoing of all that had been done, and the appointment of a practically new committee, with themselves in the prominent positions.

The plea under which this was done was, that the organization of the Congress did not properly represent the country, and that some of the New Code men of the State of New York had been appointed to office. The speciousness of this claim will be seen when it is understood that this is not a political assembly ; it is not the *country* which is to be represented, but the profession of medicine, and such men should be chosen as best typify the great profession, no matter if they were all found in one city. If the back woods of Arkansas could furnish a man who could take rank with the leading minds of medicine, he should be chosen. But if not, Arkansas should wait until it could produce such a man. As to the factional issue of new or old codes, that was a matter with which the Congress had, and desired to have, nothing whatever to do.

But the new committee assumed the reins, and proceeded to reorganize the Congress, displacing some of the men whose fame is world-wide, and who cast a lustre upon medical science, and putting in their places some of the ambitious "outs" who were great only in their own conceit. They reduced the number of sections, dropping that of dental and oral surgery, or, what is the same thing, consolidating it with general surgery. The *Medical Bulletin*, of Philadelphia, which is edited by one of the leading spirits in the revolution, says : "The omission of the section of dental and oral surgery was judicious, dentistry not being generally recognized as a legitimate department of medicine." We may well be content to rest under his ban, when he is at known issue with very much that is reputable and honorable in medicine.

Anticipating censure, the new committee fortified themselves with the opinion of lawyers concerning the legality of their action. To what end ? What have lawyers to do with the ethics of another profession ? Did the committee propose to go to extreme legal limits, or was it their desire to do what was professional and right ?

As might be imagined, their proceedings have excited a deep and indignant feeling among the better part of physicians. The protests have been many and vigorous. The most of the leading medical journals have forcibly denounced the movement. Meetings of leading medical men have been held in several of the large cities, and many of the most respectable of the appointees have announced that they will have nothing to do with the Congress as now organized. In Philadelphia, such men as Agnew, Bartholow, Brinton, Da Costa, Gross, Hays, Leidy, Mitchell, Stille, Tyson, Wood, and many others, with Yandell of Louisville, have formally withdrawn. The Philadelphia list of protestants alone includes four presidents of sections.

This action has been imitated in other cities, and so many eminent names have been withdrawn that it now seems demonstrated that, under the present organization, the Congress cannot by any possibility be made a success. The men now charged with the management of the Congress are believed to be incompetent, and they certainly do not possess the confidence of the profession, nor are they sufficiently representative of the intelligence of those for whom they assume to act. Therefore, as we said in the last number, it is probably as well that we are not involved in the quarrel. The only thing that remains is to see if the Congress cannot be rescued from the hands of those who have assumed its managment. But even this could not probably be done in time to re-reorganize with much hopes of making it what it should have been.

It is a shame and a scandal to medicine, that such a state of affairs can exist. But as long as such an organization as the American Medical Association, with its fluctuating, constantly changing membership, shall be the representative Association of American Medicine, we have little better to hope for. It is a complete political body, and, like all such associations, it is usually controlled by demagogues and wire-pullers. What medicine needs is a permanent body, that shall represent, not its tricksters and politicians, but its intelligence, its professional eminence, and its scientific attainments. This the American Association does not and cannot do, because its membership is founded upon geographical, and not upon scientific or professional representation. It now seeks to make the International Medical Congress but an enlarged session of this unrepresentative body. Should it succeed, the meeting would do well to act upon the old adage, *Ne sutor ultra crepidam;* and this for the occasion might be freely translated: "Stick the Shoemaker to his bench with his own wax."

